# The Impact of Early Intensive Behavioral and Developmental Interventions on Key Developmental Outcomes in Young Children With Autism Spectrum Disorder: A Narrative Review

**DOI:** 10.7759/cureus.92055

**Published:** 2025-09-11

**Authors:** Sravanthi Avula, Bethel T Mandefro, Sri Vidya Sundara, Xinyu Lu, Hamide Busmail, Sasika Weerakoon, Iana A Malasevskaia

**Affiliations:** 1 Pediatrics, NRI Medical College, Guntur, IND; 2 Medicine, California Institute of Behavioral Neurosciences & Psychology, Fairfield, USA; 3 Pediatrics, California Institute of Behavioral Neurosciences & Psychology, Fairfield, USA; 4 ENT, Shanghai Pudong Hospital, Shanghai, CHN; 5 Internal Medicine, California Institute of Behavioral Neurosciences & Psychology, Fairfield, USA; 6 Hospital-Based Medicine, Harvard T.H. Chan School of Public Health, Boston, USA

**Keywords:** adaptive behavior, applied behavior analysis, autism spectrum disorder, cognitive development, early intervention, early start denver model, naturalistic developmental behavioral interventions

## Abstract

Autism Spectrum Disorder (ASD) affects multiple developmental domains, and early intervention is critical for optimizing long-term outcomes. Early intensive behavioral and developmental interventions (EIBIs/EDIs), including Applied Behavior Analysis (ABA), the Early Start Denver Model (ESDM), and Pivotal Response Training (PRT), have emerged as key evidence-based strategies. This narrative review aims to evaluate the effectiveness of these interventions on cognitive, language, adaptive, and social outcomes in children under seven years of age, drawing from both primary studies and systematic reviews.

Findings from meta-analyses and primary studies indicate that EIBIs and naturalistic developmental behavioral interventions (NDBIs) are associated with significant improvements in IQ (gains of 9-15 points) and language development. However, effects on core autism symptoms are more variable. Parent-mediated and lower-intensity intervention models show promise in maintaining effectiveness while improving accessibility. Despite these encouraging outcomes, variability in study designs, outcome measures, and individual responses highlights the need for more personalized approaches.

The key gaps include limited long-term follow-up, inconsistent symptom reduction, and disparities in access. Future research should prioritize precision medicine frameworks, adaptive intervention models, and culturally responsive implementation strategies to enhance equity and maximize developmental potential for children with ASD.

## Introduction and background

Autism spectrum disorder (ASD) is a neurodevelopmental disorder characterized by deficits in social communication and the presence of restricted interests and repetitive behaviors [1]. The Centers for Disease Control and Prevention's (CDC) Autism and Developmental Disabilities Monitoring (ADDM) Network reports a prevalence of one in 31 children (3.2%) aged eight years in 2022 in the United States (US) - a significant increase from one in 36 in 2020 and one in 150 in 2000 - reflecting improved detection and broader diagnostic criteria [1,2]. ASD occurs across all racial, ethnic, and socioeconomic groups but is 3.4 times more common in boys than girls, with higher prevalence among Asian/Pacific Islander (3.8%), Black (3.7%), and Hispanic (3.3%) children compared to White children (2.7%) [2,3]. Clinically, ASD often presents in early childhood with delayed speech and language development, impaired eye contact, limited social reciprocity, restricted play, and repetitive motor behaviors.

**Figure 1:**
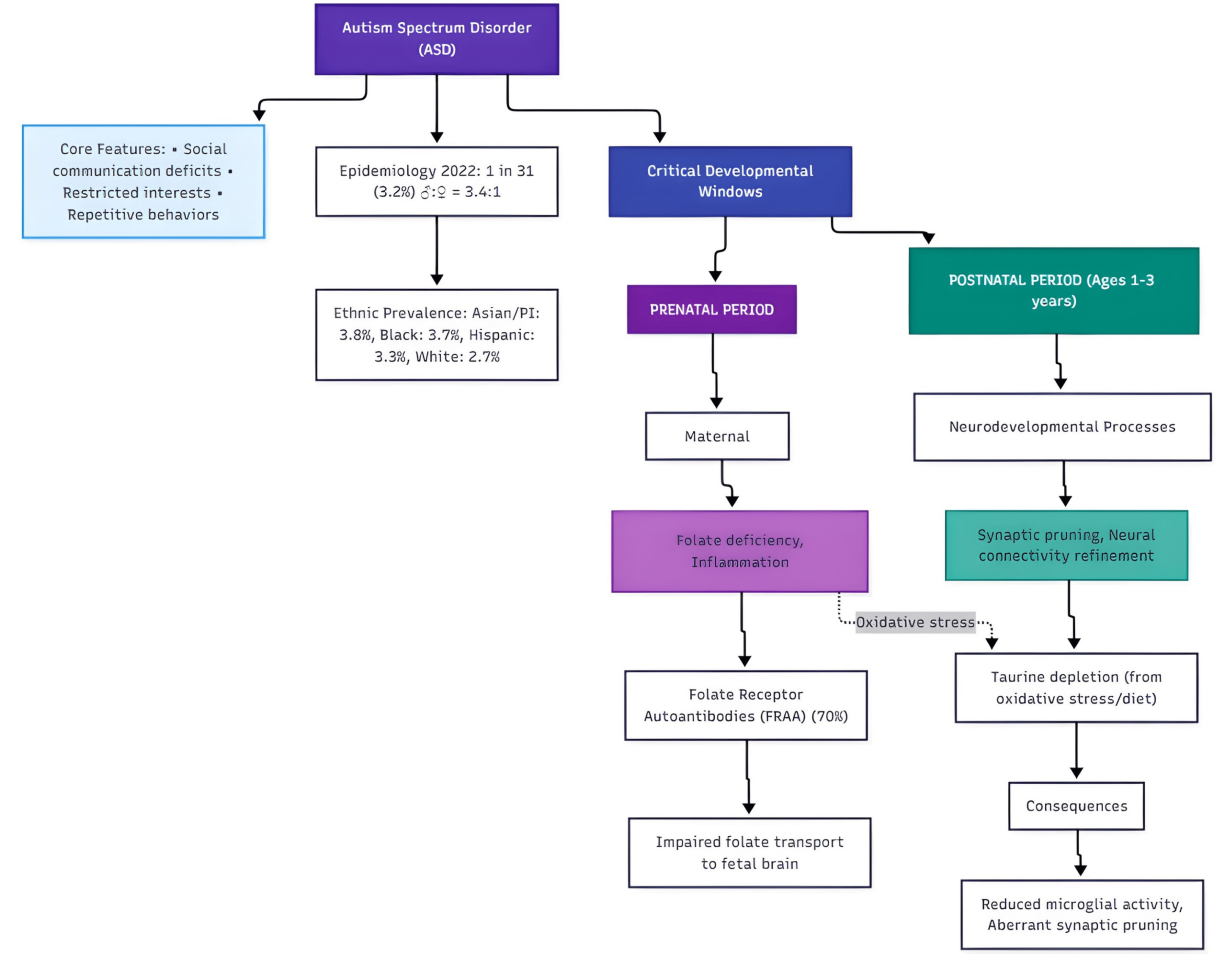
Key factors and mechanisms in autism spectrum disorder neurodevelopment Sources: [1-4] Note: Visualization created with Mermaid Live Editor by Iana Malasevskaia

Emerging research highlights two critical neurodevelopmental windows for ASD risk: (1) prenatal period, where maternal folate deficiency and inflammation may disrupt fetal brain development, and (2) early postnatal years (ages one to three), when synaptic pruning and neural connectivity are refined (Figure 1) [4]. During these windows, insufficient folate transport (due to folate receptor autoantibodies [FRAA]) and taurine depletion (from oxidative stress or dietary factors) may synergistically impair neurotypical development [4]. Notably, 70% of children with ASD exhibit FRAA, which blocks folate uptake to the brain, while low taurine levels correlate with reduced microglial activity and aberrant synaptic pruning [4]. It should be noted that these “two critical windows” remain a postulation based primarily on one study, and further evidence is needed to validate this hypothesis.

Recommended screening tools for ASD include the Modified Checklist for Autism in Toddlers, Revised with Follow-up (M-CHAT-R/F) as the primary screening tool for children aged 16-30 months (78% sensitivity, 98% specificity) [5]. For definitive diagnosis, the Childhood Autism Rating Scale (CARS) (89% sensitivity, 79% specificity) and Autism Diagnostic Observation Schedule (ADOS) (87% sensitivity, 75% specificity) are most commonly recommended, particularly for children under seven years [5]. The American Academy of Pediatrics recommends initial screening with M-CHAT-R/F at the 18- and 24-month visits, followed by confirmatory testing with ADOS or CARS when indicated [5].

Current evidence supports Early Intensive Behavioral Intervention (EIBI) as a key management approach for young children with ASD. A systematic review published in 2018 found that EIBI (20-40 hours/week for two to three years) significantly improves adaptive behavior, IQ, and language skills compared to treatment-as-usual [6]. However, the evidence quality was rated as low due to methodological limitations in the included studies, and the effects on core autism symptoms were inconsistent. These findings highlight both the potential benefits and limitations of intensive behavioral interventions.

Several critical gaps remain in our understanding of early interventions for ASD. First, the optimal intensity and duration of EIBI programs need further investigation. Second, predictors of treatment response are not well established, making it difficult to identify which children will benefit most. Third, there is limited evidence comparing EIBI to other intervention approaches. Finally, long-term outcomes beyond early childhood require more rigorous study.

This review aims to evaluate the effects of early intensive behavioral and developmental interventions (EIBIs/EDIs) on core autism symptoms, language development, cognitive abilities, and adaptive functioning in young children with ASD. By synthesizing evidence from high-quality studies, we seek to clarify the most effective intervention strategies during this critical developmental period.

## Review

Methods

This article presents a narrative review of the literature concerning EIBIs/EDIs for young children with ASD. The objective of this review is to provide a comprehensive, critical summary and synthesis of the current evidence base, drawing from both foundational and recent high-quality primary research studies and systematic reviews/meta-analyses. This approach enables a broad exploration of interventions, outcomes, and contemporary debates within the field, identifying key trends, consensus points, and remaining gaps in knowledge.

Literature Search Strategy

A comprehensive literature search was conducted to identify relevant publications utilizing several major electronic databases, including PubMed/MEDLINE, Science Direct, Europe PMC, and Google Scholar. The search strategy employed a combination of keywords and controlled vocabulary terms, such as Medical Subject Headings (MeSH) in PubMed, related to the core concepts of the review. Key search terms included variations and combinations of: "autism spectrum disorder," "ASD," "child," "toddler," "preschool," "early intervention," "early intensive behavioral intervention," "EIBI," "Applied Behavior Analysis," "ABA," "Early Start Denver Model," "ESDM," "Pivotal Response Training," "PRT," and "naturalistic developmental behavioral intervention," "NDBI." The search was initially focused on articles published within the last decade to prioritize the most current evidence, but seminal older works were also considered for foundational context. To ensure thorough coverage, the reference lists of retrieved systematic reviews, meta-analyses, and other key articles were manually examined for additional relevant studies that may not have been captured in the initial electronic search.

Study Selection and Synthesis Approach

The review prioritized including randomized controlled trials (RCTs), high-quality quasi-experimental studies, and large-scale observational studies that evaluated comprehensive early intervention models. We focused on studies where participants were children diagnosed with ASD, predominantly under seven years of age at the start of the intervention. Eligible interventions included established, evidence-based models such as Applied Behavior Analysis (ABA)-based programs, the Early Start Denver Model (ESDM), and Pivotal Response Training (PRT), particularly those described as intensive or comprehensive. The outcomes of interest were cognitive ability (IQ), language development, adaptive functioning, and core autism symptom severity, as measured by standardized assessment tools such as: the Mullen Scales of Early Learning (MSEL) for cognitive ability, the Vineland Adaptive Behavior Scales (VABS) for adaptive functioning, the Preschool Language Scale (PLS) for language development, and the Autism Diagnostic Observation Schedule (ADOS) for core symptom severity.

Given the narrative design of this review, a formal systematic data extraction or risk-of-bias assessment across all included studies was not performed. Instead, the synthesis involved a critical analysis and thematic summary of the findings from the selected literature. Evidence was organized to compare the effects of different intervention models, explore the impact of factors such as intervention intensity and delivery method, and highlight consistent findings and contradictions across the body of research. Findings from systematic reviews and meta-analyses were used to summarize the overall strength and consistency of the evidence for major conclusions.

Discussion

Models and Frameworks of Early Intervention in Autism

Early interventions (EIs) are structured therapeutic approaches designed to address the core symptoms of ASD during the critical period of early neurodevelopment. A robust body of research indicates that interventions initiated before the age of five, particularly between 18 and 36 months, yield significantly greater outcomes in cognition, language, adaptive behavior, and social communication [6-8]. The primary goal of these interventions is to leverage the brain's heightened plasticity in early childhood, thereby optimizing a child's developmental trajectory [9].

Among the most established models is EIBI, a high-intensity, therapist-led program grounded in the principles of ABA. It is typically delivered one-on-one for 20-40 hours per week over one to three years or longer. EIBI utilizes structured techniques such as discrete trial teaching, behavior shaping, and systematic reinforcement to improve cognitive and adaptive functioning. Generally initiated in toddlers as young as 18-30 months, EIBI has demonstrated substantial effects on intellectual functioning and the acquisition of daily living skills [6,9-11].

In contrast to the highly structured format of EIBI, Naturalistic Developmental Behavioral Interventions (NDBIs) have gained prominence. NDBIs integrate behavioral strategies with developmental science, emphasizing learning within natural settings like play or daily routines. Key NDBI programs include the ESDM and Pivotal Response Treatment (PRT) [12,13]. These interventions, often delivered at lower intensities (such as 10-15 hours/week), are frequently parent-mediated or therapist-guided and specifically target the development of social reciprocity, imitation, language, and shared attention [14,15].

Building on the principles of NDBIs, Parent-Mediated Interventions (PMIs) represent a critical implementation strategy. These interventions focus on training caregivers to embed therapeutic techniques into their daily interactions with their child. This approach is designed to enhance the generalization of skills from the clinic to the home environment, while also increasing accessibility and reducing the financial burden on families, particularly in low-resource settings. Parent-implemented versions of ESDM and other NDBIs have shown clear effectiveness in improving communication and reducing symptom severity [15,16].

Complementing these comprehensive behavioral and developmental frameworks are targeted therapies such as speech-language therapy and occupational therapy (OT). Speech-language therapy directly addresses deficits in expressive and receptive communication, while OT focuses on sensory integration, motor planning, and self-help skills [17,18]. These therapies are often integrated with other approaches and are typically delivered 1-2 times per week, though intensity can vary based on individual need. Studies show that dedicated speech-language therapy and OT can significantly enhance communication abilities, reduce sensory challenges, and improve a child’s participation in daily routines [19].

Ultimately, the evidence underscores that to maximize developmental outcomes, early interventions should be individualized, developmentally appropriate, and initiated as early as possible. The selection and intensity of any intervention depend on a careful consideration of the child’s age, functional level, family goals and involvement, and the availability of trained professionals [20-22].

Synthesis of Findings From Primary Research

Our analysis of the nine primary studies included in this review revealed several key insights into the effectiveness of different therapeutic approaches. The evidence demonstrated that while both behavioral and developmental interventions can produce meaningful improvements, the magnitude and consistency of these effects vary considerably by intervention type, intensity, and implementation method [12,14,15,16,18,19,23,24,25]. The findings coalesce around several critical themes regarding comparative effectiveness [12,13], the role of intervention dosage [24,25], and the importance of parent-mediated models [15,16].

Comparative effectiveness of intervention approaches: The reviewed studies suggested that naturalistic developmental interventions like ESDM show particular promise for comprehensive developmental gains. Cucinotta et al.'s (2022) direct comparison revealed that while ESDM, EIBI, and TAU all improved core autism symptoms (ADOS-2 scores), only ESDM produced significant improvements across multiple Griffiths subscales, including personal-social development (Δ = 24.37, p<0.001) and hearing/speech (Δ = 30.80, p<0.001) [23]. This aligned with Rogers et al.'s finding that ESDM provided a significant language advantage over community treatment (+4.9 months, p = 0.03) [12]. The developmental nature of ESDM, which integrates relationship-based approaches with behavioral principles, may better target the social-communication deficits central to ASD.

Dose-response considerations: These findings challenged conventional assumptions about intervention intensity. Waddington et al. (2019) demonstrated that even low-intensity ESDM (3 hours/week) could produce clinically meaningful improvements in imitation (NAP = 1.0) and functional utterances (NAP = 0.59-1.0) for preschool-aged children [24]. Similarly, Anderson et al.'s (2024) MAYAC intervention (5-10 hours/week) proved non-inferior to comprehensive behavioral intervention (≥15 hours/week) on Vineland scores (p = 0.0144) [25]. These results suggested that well-targeted, developmentally appropriate interventions may achieve comparable outcomes at lower intensities, though more research is needed to identify optimal dosing parameters.

Parent-mediated interventions: The success of parent-coaching models emerged as a consistent theme. Zhou et al.'s (2018) P-ESDM intervention (1.5 hours/week coaching) yielded significant improvements in language (23.63 vs 2.25 points, p = 0.002) and reduced parenting stress (p = 0.003) compared to those receiving community interventions [15]. Similarly, Mirenda et al. (2022) found parent coaching enhanced word understanding (p = 0.043) and quality of life (p = 0.031) more than community services alone [16]. These findings underscore the value of empowering parents as therapeutic agents, particularly for fostering generalization of skills to natural environments.

Heterogeneity of treatment response: Notable variability was seen in individual responses to intervention. While Geoffray et al. (2019) reported cognitive gains with ESDM (11.2 points, p = 0.0003), they paradoxically found decreases in social skills (p = 0.0131) and daily living skills (p = 0.0200) [14]. Similarly, Ketcheson et al.'s (2017) motor intervention improved locomotion (p<0.001) but not physical activity levels [19]. This heterogeneity highlights the need for personalized intervention approaches that account for baseline characteristics, as suggested by Rogers et al.'s (2019) finding that ADOS severity changes were moderated by baseline developmental quotient (p = 0.03) [12].

Implementation considerations: The observational data from Trajkovski et al. (2016) revealed important real-world challenges, with only 35% of families reporting satisfaction with available treatments despite 81% noting some improvement (Table 1) [18]. This disconnection underscored the importance of considering family preferences and quality-of-life outcomes alongside clinical measures, as captured in Mirenda et al.'s (2022) parent-reported outcomes [16].

**Table 1:** Review of early intervention studies for ASD ABA: Applied Behavior Analysis; ADOS: Autism Diagnostic Observation Schedule; CBI: Comprehensive Behavioral Intervention; CPRT: Classroom Pivotal Response Teaching; DQ: Developmental Quotient; ECT: Enriched Community Treatment; EIBI: Early Intensive Behavioral Intervention; ESDM: Early Start Denver Model; ITT: Intent-to-Treat; MAYAC: Modular Approach for Young Autistic Children; MPR DI: Mullen Scales of Early Learning, Daily Living Skills; NAP: Nonoverlap of All Pairs; OACIS-AS: Ohio Autism Clinical Improvement Scale - Autism Severity; PA: physical activity; PC: parent coaching; P-ESDM: Parent-Implemented Early Start Denver Model; PDDBI-P: Pervasive Developmental Disorder Behavior Inventory - Parent; RCT: randomized controlled trial; SCEI: Social Communication and Interaction; TAU: Treatment As Usual; TEACCH: Treatment and Education of Autistic and related Communication-handicapped Children; VABS: Vineland Adaptive Behavior Scales

Paper	Study design	Intervention(s)	Participants (n)	Key intervention effects
Waddington et al., 2019 [24]	Non-concurrent multiple probe (single-subject design)	Low-intensity ESDM: 3 hrs/wk for 12 wks, home-based, therapist-delivered, with no parent coaching	4	Improvements from Baseline → Intervention → Follow-up. All 4 children improved in imitation & engagement (NAP > 0.94). 3 of 4 showed large improvements in functional utterances (NAP ≥ 0.96). Skills generalized to mothers for all children
Anderson et al., 2024 [25]	Single-blind, multi-site, stratified RCT; comparative effectiveness trial	MAYAC (5-10 hrs/wk, direct therapy + parent training) vs. CBI (≥15 hrs/wk, direct therapy)	Total: 56 (MAYAC: 27; CBI: 29)	VABS: Both groups improved significantly (p < 0.0001); MAYAC was non-inferior to CBI. OACIS-AS: No significant difference in treatment responders. PDDBI-P: No significant changes for either group.
Rogers et al., 2019 [12]	Single-blind, multi-site, intent-to-treat RCT	ESDM (avg. 15 hrs/wk for 2 yrs with monthly parent coaching) vs. Community Intervention (TAU)	Total: 118 (ESDM: 55; TAU: 63)	Language: The ESDM group had a significant advantage over TAU (p = .03). No significant group differences in DQ, Autism Severity, or Adaptive Behavior. ADOS Severity effects were moderated by baseline DQ
Mirenda et al., 2022 [16]	Multi-site, stratified, parallel-design RCT with modified ITT	PC (24 wks, 1 hr/wk parent coaching) vs. ECT (24 wks of community services)	Total: 62* (PC: 32; ECT: 30)	PC group showed significantly greater gains in word understanding, quality of life, parent satisfaction, and self-efficacy (all p<0.05). No group differences in joint engagement, interaction skills, or MPR DI scores
Ketcheson et al., 2017 [19]	Non-randomized controlled pilot study	CPRT-based motor intervention (4 hrs/day, 5 days/wk for 8 wks) vs. a control group	Total: 20 (Exp: 11; Ctrl: 9)	Motor Skills: The experimental group had significant improvements (p ≤ 0.01) while the control group did not. Socialization: The experimental group showed a significant decrease in solitary play (p < 0.01). No change in PA
Trajkovski et al., 2016 [18]	Observational, descriptive internet-based survey	Survey of various interventions used by families in Macedonia (e.g., social skills training, ABA, TEACCH)	72	Parent-reported outcomes: 81% reported positive improvements. 35% were satisfied with their current treatment, while 38% were not
Geoffray et al., 2019 [14]	Prospective, multi-site observational study	ESDM: 12 hrs/wk (10 hrs clinic, 2 hrs natural environment) with parent coaching and family workshops	19	Significant improvements in overall cognitive level (p = 0.0003) and receptive language DQ. A significant decrease was reported in social skills (p = 0.0131) and daily living skills (p = 0.0200)
Zhou et al., 2018 [15]	Non-randomized controlled trial	P-ESDM (1.5 hrs/wk parent coaching for 26 wks) vs. Community Intervention (TAU)	Total: 58 (P-ESDM: 30; TAU: 28)	P-ESDM group showed greater improvement than TAU in general development, language (p = 0.002), eye-hand coordination (p=0.026), social affect, and parent-reported skills. Parenting stress decreased in the P-ESDM group
Cucinotta et al., 2022 [23]	Retrospective, observational chart review	Comparison of three groups (6 hrs/wk for 1 yr): ESDM vs. EIBI vs. TAU (non-specialized therapy)	Total: 90 (ESDM: 41; EIBI: 13; TAU: 36)	ADOS-2 Severity: All three groups improved equally and significantly (p<0.001). Griffiths Scales: Only the ESDM group showed significant improvements across multiple domains (e.g., locomotor, personal-social, hearing/speech)

Synthesis of Findings From Published Systematic Reviews and Meta-Analyses

Efficacy of early interventions on core outcomes: The synthesized evidence from 11 meta-analyses demonstrates that EIBIs and NDBIs yield significant improvements in key developmental domains for young children with ASD. Cognitive abilities, as measured by IQ scores, showed the most consistent gains, with EIBI programs reporting mean increases of 9.16 - 15.44 points [6,9]. Similarly, NDBIs produced moderate cognitive effects (g = 0.48 - 0.76), particularly in studies applying the Early Start Denver Model [8,13]. Language outcomes followed this trend, with expressive and receptive skills improving under both EIBI (SMD = 0.51 - 0.55) and NDBI approaches (g = 0.28 - 0.32), though effect sizes varied by intervention intensity and delivery method [17]. Adaptive behavior, assessed via VABS, also improved significantly under EIBI (MD = 7.00-9.58), albeit with smaller effects in community-based settings [20].

In contrast, reductions in core ASD symptoms-such as social communication deficits and restricted/repetitive behaviors-were less robust. While NDBIs demonstrated moderate social communication benefits (g = 0.35-0.65), EIBI effects were inconsistent across reviews (SMD = -0.34 to -0.68; [6,8]. This discrepancy may reflect differences in intervention targets: EIBIs prioritize discrete skill acquisition, whereas NDBIs emphasize socially embedded learning. Notably, no intervention category showed significant effects on parental stress or long-term symptom severity, underscoring gaps in holistic outcome measurement [10,11] (Table 2).

**Table 2:** Synthesis of recent systematic reviews and meta-analyses on early intensive behavioral and developmental interventions for young children with ASD ASD: autism spectrum disorder; ABA: Applied Behavior Analysis; EIBI: Early Intensive Behavioral Intervention; EDI: Early Developmental Intervention; ESDM: Early Start Denver Model; PRT: Pivotal Response Training; DTT: Discrete Trial Training; PECS: Picture Exchange Communication System; NDBI: Naturalistic Developmental Behavioral Intervention; TAU: Treatment as Usual; SST: Social Skills Training; AAC: Augmentative and Alternative Communication; TEACCH: Treatment and Education of Autistic and Communication Handicapped Children; DSP: Developmental Social-Pragmatic models; PMI: Parent-Mediated Interventions; ADOS: Autism Diagnostic Observation Schedule; CARS: Childhood Autism Rating Scale; PLS: Preschool Language Scales; CELF: Clinical Evaluation of Language Fundamentals; EVT: Expressive Vocabulary Test; PPVT: Peabody Picture Vocabulary Test; VABS: Vineland Adaptive Behavior Scales; ABAS: Adaptive Behavior Assessment System; RCT: randomized controlled trial; QED: quasi-experimental design; CCT: controlled clinical trial; DSM: Diagnostic and Statistical Manual of Mental Disorders; ICD: International Classification of Diseases; MD: mean difference; ES: effect size; SMD: standardized mean difference; g: Hedges' g; CI: confidence interval

Study - citations	Intervention	Outcome measured	Intervention effects	Future research
Reichow et al., 2018 [6] - 114 citations	EIBI, NDBI, SST, AAC, PECS	Adaptive behavior: VABS Composite, Autism symptom severity	Adaptive behavior: EIBI improved by MD 9.58 (95% CI 5.57 to 13.60, P < 0.0001) Autism symptom severity: EIBI reduced by SMD -0.34 (95% CI -0.79 to 0.11, P = 0.14) IQ: EIBI improved by MD 15.44 (95% CI 9.29 to 21.59, P < 0.001) Expressive language skills: EIBI improved by SMD 0.51 (95% CI 0.12 to 0.90, P = 0.01) Receptive language skills: EIBI improved by SMD 0.55 (95% CI 0.23 to 0.87, P = 0.001) Problem behavior: EIBI reduced by SMD -0.58 (95% CI -1.24 to 0.07, P = 0.08)	Additional studies using rigorous research designs are needed to strengthen conclusions about EIBI's effects. More studies are required in Korea to generalize EIBI's effectiveness. Better experimental designs are needed to assess PMI's effectiveness, particularly in Korea. Further research is needed to establish the effectiveness of P-ESDM and TEACCH. Newly introduced interventions require further research to establish their evidence base. Future research should leverage advancements in technology and data collection for personalized assessments and treatments
Shi et.al., 2021 [8] - 20 citations	EIBI, ESDM	IQ, language, behavioral (adaptive functioning and symptomatology)	IQ improvement: ESDM: ES = 1.37 (95% CI: 0.95 to 1.80) EIBI: ES = 0.53 (95% CI: 0.16 to 0.90) Symptom reduction: EIBI: ES = -1.27 (95% CI: -1.96 to -0.58) Expressive language: ES = 1.12 (95% CI: 0.70 to 1.53) Receptive language: ES = 1.11 (95% CI: 0.83 to 1.40) ASD symptom severity: ES = -0.68 (95% CI: -1.24 to -0.12) Communication: ES = 0.75 (95% CI: 0.47 to 1.02) Social skills: ES = 0.55 (95% CI: 0.17 to 0.92) Daily living skills: ES = -0.05 (95% CI: -0.49 to 0.39) Composite score: ES = 0.15 (95% CI: -0.28 to 0.57) Adaptation composite scores: ES = 0.47 (95% CI: 0.11 to 0.83)	Increase understanding of outcomes in childhood to inform effective school curriculum and targeted support. Investigate outcomes post-middle childhood (5 years and later). Conduct secondary research on outcomes in 5–18-year-old children. Emphasize empirical studies to determine long-term effects. Use randomized controlled trials. Record specific intervention approaches and monitor fidelity. Collect detailed information on education and intervention strategies during mid-childhood and adolescence. Explore ESDM programs for lower- and higher-functioning ASD. Focus on follow-up measurement and comprehensive initial measurement
Daniolou et al., 2022 [7] - 16 citations	ABA, DSP	Cognitive ability, language skills, communication, socialization, adaptive behavior, daily living skills, motor skills	Cognitive ability: g = 0.32; 95% CI: 0.05, 0.58; p = 0.02 (significant) Daily living skills: g = 0.35; 95% CI: 0.08, 0.63; p = 0.01 (significant) Motor skills: g = 0.39; 95% CI: 0.16, 0.62; p = 0.001 (significant) Expressive language: g = 0.10; 95% CI: -0.00, 0.20; p = 0.06 (non-significant) Receptive language: g = 0.12; 95% CI: -0.06, 0.31; p = 0.19 (non-significant) Communication: g = 0.06; 95% CI: -0.07, 0.12; p = 0.36 (non-significant) Socialization: g = 0.10; 95% CI: -0.06, 0.27; p = 0.21 (non-significant) Adaptive behavior composite: g = 0.20; 95% CI: -0.16, 0.55; p = 0.27 (non-significant) After excluding studies with bias: Cognitive ability: g = 0.25; 95% CI: -0.04, 0.54; p = 0.09 (non-significant) Daily living skills: g = 0.28; 95% CI: 0.04, 0.52; p = 0.02 (significant) Motor skills: g = 0.49; 95% CI: 0.28, 0.79; p ≤ 0.00001 (significant)	Ensure participants attain sufficient intervention dosage, as high-intensity interventions have shown positive outcomes. Design or use more sensitive measures than standardized ones. Examine longer-term effects through follow-up studies. Create specific intervention groups with comparable cognitive abilities and smaller age ranges to explore subgroup responses. Conduct studies that meet high-quality research standards to draw valid and accurate conclusions
Rodgers et al., 2021 [9] - 21 citations	ABA, EIBI, NDBI	Cognitive ability (intelligence quotient) and adaptive behavior (VABS)	VABS Scale: Early intensive ABA-based interventions: MD = 7.00 (95% CI: 1.95–12.06). Cognitive ability (intelligence quotient): Early intensive ABA-based interventions: MD = 14.13 (95% CI: 9.16–19.10)	Investigate which supports and interventions are most effective for children and families, prioritizing outcomes meaningful to the autism community and longer-term follow-up. Conduct high-quality research studies to address concerns about internal validity and effectiveness. Evidence - Prioritize relevant outcomes and use reliable outcome measures in future intervention evaluation trials. Consider the length of follow-up to understand the durability of early benefits. Conduct high-quality comparative research studies to estimate the cost-effectiveness of early interventions
Tiede et al., 2019 [13] - 102 citations	NDBI	Expressive language, reduction in symptoms of autism spectrum disorder, play skills, social engagement, overall cognitive development, joint attention, and receptive language	Expressive language: g = 0.32 (significant) Reduction in symptoms of autism spectrum disorder: g = -0.38 (significant) Play skills: g = 0.23 (significant) Social engagement: g = 0.65 (significant) Overall cognitive development: g = 0.48 (significant) Joint attention: g = 0.14 (marginal) Receptive language: g = 0.28 (marginal)	Examine how child characteristics /moderators (like age and symptom severity) affect outcomes. Identify which parts of the intervention are most effective. Compare NDBI to other active, evidence-based interventions (e.g., EIBI, PECS)
Sandbank et al., 2019 [21] - 353 citations	ABA, ESDM, TEACCH	Core features of ASD: social communication, restricted/repetitive patterns of behaviors, interests, or activities, sensory. Related outcomes: language, motor, adaptive, cognitive, academic, play, sleep, brain imaging, social emotional/challenging behavior	Behavioral: Adaptive: 0.24-0.46 (significant); Cognitive: 0.24-0.46(significant); Language: 0.24-0.46 (significant); Motor: 0.24-0.46 (significant); Social: 0.24-0.46 (significant); Behaviors: 0.24-0.46 (significant); Symptoms: 0.24-0.46 (significant) Developmental: Language:0.06 (Nonsignificant); Social: 0.30 (significant) NDBI: Adaptive: -0.01-0.35 (significant); Cognitive: -0.01-0.35 (significant); Language: -0.01-0.35 (significant); Play: -0.01-0.35 (significant); RRB: -0.01-0.35 (significant); Social: -0.01-0.35 (significant); Behaviors: -0.01-0.35 (significant); Symptoms: -0.01-0.35 (significant) Sensory: Language: 0.28 (Nonsignificant) TEACCH: Social: -0.11 (Nonsignificant) Tech-based: Social: 0.05 (Nonsignificant); Behaviors: 0.42 (Nonsignificant)	Conduct high-quality studies with randomized trials and independent assessors. Develop new, low-cost measures sensitive to change. Provide detailed descriptions of measures and assessment processes. Develop manualized protocols for interventions. Publish unadjusted means and standard deviations. Include paired proximal and distal measures. Conduct mediation analyses to understand developmental pathways
Eckes et al., 2023 [10] - 31 citations	ABA	Adaptive behavior: VABS, intellectual functioning, language abilities, symptom severity, and parental stress	Intellectual functioning: Comprehensive ABA-based interventions (SMD = 0.51, 95% CI [0.09; 0.92]) Adaptive behavior: Comprehensive ABA-based interventions (SMD = 0.37, 95% CI [0.03; 0.70]) Language abilities: No significant difference between treatment and control groups Symptom severity: No significant difference between treatment and control groups Measures of caregiver stress showed no notable differences between intervention and control groups in the analyzed studies	Conduct more methodologically sound studies to validate the effectiveness of comprehensive ABA-based interventions for ASD. Use alternative statistical approaches like growth curve analyses to develop personalized treatment options. Develop and evaluate better diagnostic procedures to reduce biases in meta-analyses. Address ethical concerns in RCTs by comparing helpful interventions or using adaptive rolling designs
Nahmias et al., 2019 [20] - 103 citations	EIBI, TAU	Cognitive functioning, communication functioning, social functioning, and adaptive behavior functioning	Cognitive: Model EI programs had moderate gains; TAU and variable EI had small gains (p < .05) Communication: Hedges's g ranged from -0.26 to 0.70; average effect size was small (p < .001) Social: Hedges's g ranged from -0.96 to 0.75; average effect size was small Adaptive Behavior: Hedges's g ranged from -1.25 to 0.95; average effect size was small (p < .001) Intervention duration was negatively associated with communication and adaptive behavior outcomes. Programs associated with universities and hospitals had significantly better outcomes than other community programs	Developing strategies for the wide-scale implementation of effective EI based on understanding community-based EI effectiveness. Investigating ongoing treatment monitoring to adapt or change interventions based on observed benefits. Exploring public-academic collaborations to improve community practice. Addressing limitations in the intervention model and participant characteristics to provide more comprehensive insights
Hampton et al., 2016 [17] - 106 citations	Parent + Clinician	Spoken-language outcomes	Overall mean effect size of early intervention on spoken-language outcomes: g = 0.26 (CI = 0.11 to 0.42); significant. Interventions delivered by clinician and parent: greater gains than those delivered by clinician or parent only	Future research should report standard language measures as well as child (cognitive ability and socio-economic status) and intervention characteristics to improve evidence related to the effects of interventions on spoken communication in children with ASD
Rodgers et al., 2020 [11] - 43 citations	ABA	Adaptive behavior (VABS score). IQ. Language development. Autism symptom severity. Presence of behaviors that challenge. Placement into mainstream or specialist schools	Adaptive behavior after 2 years: Early intensive ABA-based interventions: mean difference 7.00 (95% CI 1.95 to 12.06) Treatment as usual/eclectic interventions: baseline Cognitive ability (intelligence quotient): After 1 year: mean difference 9.16 (95% CI 4.38 to 13.93) After 2 years: mean difference 14.13 (95% CI 9.16 to 19.10) Language development: no statistically significant effects found. Autism symptom severity: no clear evidence of effect. Challenging behaviors: no statistically significant effects found. Adverse effects: rarely recorded	Further studies on early intensive ABA interventions with well-defined comparators and relevant outcomes. Identifying effective components of ABA interventions. Evaluating outcomes meaningful to autistic children and families, including adverse effects. Establishing standardized outcomes and involving the autism community. Using diverse methodologies to address long-term effects and evidence gaps. Identifying interventions with strong clinical and cost-effectiveness evidence. Addressing methodological limitations in previous studies
Wergeland et al., 2022 [22] - 14 citations	ABA, EIBI	Adaptive behavior, cognition, communication, and socialization	Adaptive behavior: g = 0.81 (significant) Cognition: g = 0.76 (significant) Communication: g = 1.27 (significant) Socialization: g = 0.81 (significant) Average effect size at post-treatment: g = 0.94 (significant) Average effect size at follow-up: g = 1.08 (significant)	More detailed classification of programs based on core components. Common standard of outcome measures for assessing effectiveness. Including more detailed characteristics of children and their families in studies. Further implementation of evidence-based interventions in routine clinical care
Pan et al., - 23 citations	EIBI, ESDM, PRT	Social communication, adaptive behavior, play, language, social, emotional, or challenging behavior. Measures of diagnostic characteristics of autism	Behavioral interventions on social emotional or challenging behavior outcomes: Hedges' g = 0.58, 95% CI 0.11 to 1.06; P = 0.02. Developmental interventions on social communication: Hedges' g = 0.28, 95% CI 0.12 to 0.44; P = 0.003 Naturalistic developmental behavioral interventions: Adaptive behavior: Hedges' g = 0.23, 95% CI 0.02 to 0.43; P = 0.03 Language: Hedges' g = 0.16, 95% CI 0.01 to 0.31; P = 0.04 Play: Hedges' g = 0.19, 95% CI 0.02 to 0.36; P = 0.03 Social communication: Hedges' g = 0.35, 95% CI 0.23 to 0.47; P<0.001 Measures of diagnostic characteristics of autism: Hedges' g = 0.38, 95% CI 0.17 to 0.59; P = 0.002 Technology-based interventions: Social communication: Hedges' g = 0.33, 95% CI 0.02 to 0.64; P = 0.04 Social emotional or challenging behavior outcomes: Hedges' g = 0.57, 95% CI 0.04 to 1.09; P = 0.04 Excluding caregiver or teacher report outcomes: Developmental interventions on social communication: Hedges' g = 0.31, 95% CI 0.13 to 0.49; P = 0.003 NDBIs on social communication: Hedges' g = 0.36, 95% CI 0.23 to 0.49; P<0.001 NDBIs on measures of diagnostic characteristics of autism: Hedges' g=0.44, 95% CI 0.20 to 0.68; P = 0.002 Excluding high risk of detection bias: NDBIs on measures of diagnostic characteristics of autism: Hedges' g = 0.30, 95% CI 0.03 to 0.57; P = 0.03	Prioritize randomized controlled trials over quasi-experimental designs to establish intervention efficacy. Develop standardized procedures for monitoring and reporting adverse events, adverse effects, and harms. Conduct long-term follow-up studies to assess sustained negative impacts of interventions. Develop shared definitions and monitoring procedures for adverse events tailored by intervention type

Comparative effectiveness of intervention approaches: When comparing intervention frameworks, EIBI/ABA programs exhibited stronger effects on cognitive and adaptive outcomes but required high intensity (20-40 hours/week) to achieve clinically meaningful gains [7]. Parent-mediated interventions, though less intensive, demonstrated promise for language development (g = 0.26), particularly when combining clinician and caregiver delivery [16].

NDBIs emerged as a balanced alternative, with moderate effects across cognitive (g = 0.48), language (g = 0.32), and social domains (g = 0.65) [13]. Their naturalistic approach may enhance generalization of skills to everyday contexts, a noted limitation of structured EIBI protocols [21]. However, head-to-head comparisons between EIBIs and NDBIs remain scarce, and their cost-effectiveness is poorly understood [11].

Summary of evidence for early interventions in ASD: The synthesis of previous systematic reviews and meta-analyses revealed several key findings regarding early interventions for ASD. EIBI/ABA-based interventions demonstrated robust efficacy for improving cognitive abilities, language skills, and adaptive behavior, with multiple studies reporting statistically significant effect sizes. However, these interventions showed inconsistent results for core ASD symptoms, including social communication deficits and restricted/repetitive behaviors.

In contrast, NDBIs exhibited more comprehensive benefits across developmental domains, with particular strength in enhancing social communication skills - a core challenge in ASD. Developmental interventions showed more selective effects, primarily benefiting social communication outcomes. Parent-mediated and technology-based approaches demonstrated promising but less consistent results, highlighting the need for further research.

These findings suggest that while EIBI/ABA remains effective for skill-building, NDBIs may offer broader benefits for core ASD symptoms (Table 3).

**Table 3:** Heatmap: summary of evidence for early interventions in ASD 🟢 Consistent positive effect: most reviews report significant, positive outcomes. 🟡 Mixed/inconsistent effect: reviews report conflicting or non-significant findings. ⚪ Limited/no evidence: this combination was not sufficiently studied in the reviewed papers EIBI: Early Intensive Behavioral Intervention; ABA: Applied Behavior Analysis; NDBI: Naturalistic Developmental Behavioral Intervention; ASD: autism spectrum disorder

Intervention category	Cognitive skills	Language skills	Adaptive behavior	Social communication	ASD symptom severity
Behavioral (EIBI/ABA)	🟢	🟢	🟢	🟡	🟡
NDBI	🟢	🟢	🟢	🟢	🟢
Developmental	⚪	🟡	⚪	🟢	⚪
Parent-Mediated	🟡	🟢	🟡	🟡	⚪
Technology-Based	⚪	⚪	⚪	🟡	🟡

Limitations and Methodological Challenges

Several limitations temper the interpretability of these findings. First, methodological heterogeneity-including variability in outcome measures (e.g., ADOS vs. VABS), intervention dosages, and control conditions-complicates cross-study comparisons [7,21]. Second, only four reviews addressed fidelity of implementation [22], raising concerns about whether reported effects reflect real-world applicability. Third, the underrepresentation of girls, ethnic minorities, and children with co-occurring intellectual disabilities limits generalizability [20]. Finally, publication bias toward positive results may inflate efficacy estimates, as noted in meta-analyses with small-study effects [10].

Strengths and Limitations of the Current Review

This literature review provides a comprehensive synthesis of evidence on early interventions for ASD, integrating findings from both primary studies and systematic reviews/meta-analyses. Among its key strengths is the systematic comparison of intervention approaches (EIBI, NDBIs, parent-mediated models), which clarifies their relative efficacy across cognitive, language, adaptive, and social domains. The inclusion of recent high-quality meta-analyses strengthens the validity of conclusions [6,7,8,9,13], while the focus on neurodevelopmental timing (ages 1-5 years) aligns with current understanding of critical windows for intervention [4]. Additionally, the review highlights practical implementation factors, such as parent coaching and low-intensity models, which are crucial for real-world applicability [15,16,24,25].

However, several limitations must be acknowledged. First, the review is narrative rather than systematic, which may introduce selection bias in study inclusion. While major meta-analyses are covered, some individual studies or newer interventions (e.g., telehealth-delivered therapies) may have been overlooked. Second, the heterogeneity of outcome measures across studies (e.g., ADOS for core symptoms vs. Vineland for adaptive behavior) complicates direct comparisons of effect sizes [6, 7, 21]. Third, the review does not quantitatively synthesize results, relying instead on qualitative trends from existing meta-analyses. Finally, while disparities in access are noted, the review could further explore cultural and socioeconomic barriers to intervention uptake, particularly in low-resource settings [18,20].

Future research directions in early intervention in autism

The field of early autism intervention must advance toward precision medicine approaches to address the well-documented heterogeneity in treatment response. Future studies should integrate biomarkers - including genetic profiles, neural connectivity patterns, and metabolic markers - to predict which children will benefit most from specific interventions [4]. Leveraging artificial intelligence (AI) and machine learning to analyze these complex, multi-modal datasets represents a pivotal next step, enabling the development of sophisticated predictive models that can match individual child profiles to optimal intervention pathways. For instance, research could examine whether children with particular neurobiological profiles respond better to naturalistic developmental interventions versus structured behavioral approaches. Adaptive intervention designs that dynamically adjust intensity based on ongoing progress monitoring should be tested, building on findings that baseline developmental levels moderate treatment effects [12]. AI-driven algorithms can power these adaptive systems, using real-time data on child progress to personalize intervention dosage and strategies dynamically. Such personalized approaches would move beyond current one-size-fits-all models to optimize outcomes while reducing unnecessary resource expenditure.

Implementation science must guide the optimization of intervention delivery in real-world settings. Priority should be given to comparative effectiveness trials that directly contrast leading intervention models while systematically testing different delivery formats (e.g., clinician-led vs. parent-mediated) and intensities. These studies should incorporate rigorous cost-effectiveness analyses and standardized fidelity measures, while expanding outcome assessment to include family-centered metrics like quality of life and treatment satisfaction [11]. Here, AI-powered tools can revolutionize implementation by automating the assessment of treatment fidelity through video or audio analysis of sessions, providing objective, scalable data on intervention quality. Emerging technologies, including AI-assisted coaching and virtual reality platforms, show promise for increasing accessibility but require robust evaluation. Concurrently, research must address persistent disparities by investigating cultural adaptations of evidence-based practices and systematically examining barriers faced by underrepresented groups in accessing services [20]. AI can also help bridge this gap by powering low-cost, accessible telehealth platforms that deliver personalized support and training to families in remote or underserved areas, ensuring equitable access to effective care worldwide.

Finally, the field needs longitudinal mechanistic studies to understand both the durability and neurobiological foundations of intervention effects. Research must follow participants into adolescence and adulthood to determine whether early gains translate into meaningful improvements in independence, employment, and mental health. Multimodal studies combining behavioral measures with neuroimaging and molecular analyses could reveal how different therapies modify neural circuitry, and whether these neural changes mediate clinical outcomes [4]. Advanced AI analytics will be crucial for interpreting the vast, complex datasets generated by these longitudinal studies, identifying patterns and predictors of long-term success that are beyond the scope of traditional statistical methods. The potential synergy between behavioral interventions and biologically targeted approaches (e.g., nutritional supplementation for metabolic subgroups) represents another promising avenue. Together, these directions will transform autism intervention from a generic approach to a truly personalized model of care that maximizes outcomes across the lifespan.

## Conclusions

EIBIs/EDIs demonstrate measurable benefits across core developmental domains in children under seven with ASD. High-intensity, structured programs - especially those initiated before the age of three - consistently support improvements in cognitive functioning, language acquisition, and adaptive behavior. While evidence for reductions in core autism symptoms is mixed, the collective findings strongly endorse early access to individualized, high-quality intervention. Parent-mediated and community-based models show promise in improving scalability and accessibility, especially in low-resource settings. Future research should focus on long-term outcomes, precision-based tailoring of intervention models, and culturally adapted delivery mechanisms to expand the reach and impact of early intervention globally.
